# Hypotensive, Angiotensin Converting Enzyme (ACE) Inhibitory and Diuretic Activities of the Aqueous-methanol Extract of *Ipomoea reniformis*

**Published:** 2013

**Authors:** Qaiser Jabeen, Naveed Aslam

**Affiliations:** Faculty of Pharmacy and Alternative Medicine, Department of Pharmacy, the Islamia University of Bahawalpur, Pakistan.

**Keywords:** *Convolvulus reniformis*, *Evolvulus emarginatus*, *Merremia emearginata*, Antihypertensive, Diuretic

## Abstract

*Ipomoea reniformis *Roxb. (Convolvulaceae) is a small, weedy herb used for the management of cardiac problems in traditional systems of medicine in India and Pakistan. Objective of the present study was to investigate the hypotensive, diuretic and angiotensin converting enzyme (ACE) inhibitory activities of the aqueous-methanol (30:70) crude extract of the dried aerial parts of *I. reniformis *(Ir.Cr.) in rats.

To record blood pressure lowering effects of the Ir.Cr, different doses of the extract were administered through jugular vein to the ketamine-diazepam anesthetized normotensive rats and blood pressure was recorded via carotid artery. ACE inhibitory activity of the extract was studied *in-vitro*; using hippuryl-l-histidyl-l-leucine as substrate, the product hippurate was quantified spectrophotometrically after reacting with cyanuric chloride/dioxane reagent. Effects of intraperitoneal administration of the extract on urine and urinary electrolyte excretion were also investigated in rats.

The extract (Ir.Cr.) produced 21.51 ± 3.41, 28.99 ± 2.30, 53.34 ± 0.88 and 61.71 ± 3.37% fall in mean arterial blood pressure of the anesthetized rats at the doses of 0.1, 0.3, 1.0 and 3.0 mg/Kg, respectively. Ir.Cr. was found to have serum ACE inhibitory activity, with IC_50 _value of 422 ± 21.16 μg/mL. The extract also increased urine volume and urinary Na^+ ^excretion significantly at the doses of 30 and 50 mg/Kg in rats. The study concludes that the crude extract of *Ipomoea reniformis *(Ir.Cr.) has hypotensive, ACE inhibitory and diuretic activities, which provide the scientific justification for the traditional uses of the plant as cardioprotective, antihypertensive and diuretic remedy.

## Introduction

High blood pressure is the leading manageable risk factor for morbidity and mortality due to various cardiovascular diseases, such as myocardial infarction, heart failure, stroke and renal damage. Overall, 26.4% of world’s adult population had hypertension in 2000, and the percentage is predicted to increase by about 60%; *i.e*. to 29.2% of total adult population by the year 2025 (1). Worldwide, about 7.6 million of premature deaths and 92 millions disability adjusted life years (DALYs) are attributed to hypertension. About 80% of the hypertension burden is seen in middle-aged patients from low and middle income groups (2). Epidemiological studies have shown that normalization of blood pressure is associated with significant reduction in major cardiovascular events (3). In developing countries, majority of population uses botanical drugs due to easy access and affordability (4). Several botanicals have been used for the treatment of hypertension. One of such plants is *Ipomoea reniformis.*

*Ipomoea reniformis *Roxb. belongs to the plant family Convolvulaceae. It is a small creeping, perennial, weedy herb, that grows in damp places of fields, roadsides and forest floors. It is also called as *Convolvulus reniformis *Roxb., *Evolvulus emarginatus *Burm. f., *Merremia emearginata *(Burm. f.) Hallier f. and *Merremia gangetica *Auct.. The herb is distributed in the Subcontinent, China, Indonesia, Australia and Africa (5). In India and Pakistan, the herb is used in food and in traditional systems of medicine for the treatment of sexual debility (6), stomach problems (7), epilepsy, leucoderma, cough, headache, neuralgia, rheumatism, inflammation, fever, kidney and bladder diseases, heart problems and anemia. The herb is also used as diuretic and laxative (8-10).

Few scientific studies have reported the phytochmeical and pharmacological aspects of the herb. Resins, glycosides, reducing sugars, amino acids, tannins and fixed oils have been reported to be present in various extracts of the plant. Seeds of the plant have shown to be rich in caffeic, p-coumaric, ferulic and sinapic acids esters (10). Pharmacological investigations have demonstrated that the herb has antioxidant, *α*-amylase inhibitory (11), nephroprotective (12), anti-inflammatory (13) and anticancer (14) activities. Reports on scientific evidences for blood pressure lowering and cardiovascular protective effects of the *Ipomoea reniformis *are scarce and/or are inadequately demonstrated. Therefore, we studied the aqueous-methanol extract of the aerial parts of the herb for hypotensive effects in rats. Possibilities of involvement of serum ACE inhibitory and diuretic activities contributing to blood pressure lowering effect were also investigated in the present study.

## Experimental

*Animals*

Sprague-Dawley rats of either sex weighing between 250-300 g and albino mice of either sex weighing between 15-30 g were used in the experiments. Animals were housed at the animal house of the department of Pharmacy, the Islamia University of Bahawalpur, Pakistan, kept in housing cages with saw dust (renewed after every 48 h), maintained at temperature of 25 ± 3°C and exposed to 12 h light/12 h dark cycles. The animals had free access to water and a standard diet. The “Guide for the care and use of laboratory animals” issued by Institute of Laboratory Animal Research, Commission on Life Sciences, National Research Council (1996) was complied with and the study was approved by the Ethical Committee of the faculty of Pharmacy and Alternative Medicine, the Islamia University of Bahawalpur.

*Plant material*

The dried aerial parts of *Ipomoea reniformis *were purchased from a reputed local herbal shop, i.e. Shadab Dawakhana, Shahi Bazar, Bahawalpur, Pakistan, and identified by Mr. Abdul Hameed, Botanist of the Cholistan Institute of Desert Studies, the Islamia University of Bahawalpur. A sample of the plant material was kept in the herbarium of the Pharmacology Section, faculty of Pharmacy and Alternative Medicine, the Islamia University of Bahawalpur, and voucher No. IR-AP-08-10-012 was assigned to it for future reference.

*Chemicals*

Acetylcholine, cyanuric chloride, dimethyl sulphoxide (DMSO) and hippuryl-l-histidyl-l-leucine (HHL) were purchased from Sigma-Aldrich, USA. Folin-Ciocalteu reagent was purchased from Merck, Germany. Ketamine injections (Ketalar^®^, Akahai Pharmaceuticals, Karachi), diazepam injections (Valium^®^, Roche Pharmaceuticals, Karachi), adrenaline injections (PDH Pharmaceuticals, Lahore), heparin injections (Heparol^®^-5000, China), captopril (Bristol-Myers Squibb, Pakistan) and frusemide injections (Lasix^®^, Sanofi-Aventis, Karachi) were made available from the drug store. All other chemicals/solvents were of analytical grade.

*Extraction procedure*

The plant material was ground in an electric grinder to a coarse powder and soaked in aqueous-methanol (30: 70) at room temperature with occasional stirring for three days. It was filtered through muslin cloth and then through Whatman grade 1 filter paper. The procedure of soaking and filtration was repeated with the residue using fresh solvent for two more times. All the three filtrates were combined and evaporated using rotary evaporator (Heidolph Laborota-efficient-4000, Germany) under reduced pressure at temperature ranging between 40-50°C to a thick, semisolid paste of greenish brown colour; *i.e. *the crude extract of *Ipomoea reniformis *(Ir.Cr.). The yield of the extract was 11.9% (w/w). The extract was solubilized in normal saline containing 5% DMSO for blood pressure experiments, in 50% DMSO for ACE inhibitory assay and suspended in normal saline for diuretic and toxicity assays.

*Phytochemical analysis*

Quantitative determination of total phenolic contents was performed by Folin-Ciocalteu method using gallic acid as standard as described by Chang *et al*. (15). Preliminary qualitative screening of major secondary metabolites for presence of alkaloids, saponins, flavonoids, tannins, anthraquinones, cyanogenic glycosides and coumarins was conducted by the standard methods already described by various authors (16-18).

*Blood pressure measurements in anesthetized rats*

The blood pressure (BP) of the anesthetized rats was recorded by method described by Gilani *et al. *(19) with some modifications. Animals were anesthetized with ketamine (50-80 mg/Kg) and diazepam (5 mg/Kg), both injected intraperitoneally through separate syringes. This anesthetic combination has been shown to provide adequate anesthesia in rats with stable cardiovascular parameters within normotensive limits (20, 21). 

Animals were fixed in supine position on a dissecting table. Temperature was maintained with the help of an overhead lamp. Trachea, right jugular vein and left carotid artery were exposed by a small mid-tracheal incision. The trachea was cannulated with 18 gauge polyethylene tubing (outer diameter 1.27 mm, internal diameter 0.84 mm) to facilitate spontaneous respiration. The right jugular vein was cannulated with polyethylene tubing PE-50 (outer diameter 0.97 mm, internal diameter 0.58 mm) for intravenous injection of drugs and the plant extract solutions. The left carotid artery was cannulated with polyethylene tubing PE-50 filled with heparinized saline (60 IU/mL) and connected to a pressure transducer (MLT0699 disposable BP transducer, AD Instruments, Australia) filled with the same solution. The pressure transducer was coupled with PowerLab 4/30 and LabChart Pro software (AD Instruments, Australia) for BP and heart rate (HR) recordings. A system calibration was performed with the help of mercury manometer connected to pressure transducer before the start of first experiment every day. The exposed surface of the cannulation area was covered with a piece of cotton swab moistened with warm saline. Heparinized saline (0.1 mL) was injected to cannulated rat to prevent blood clotting. Acetylcholine (1 μg/Kg) and adrenaline (1 μg/Kg) were used to check the hypotensive and hypertensive responsiveness of each animal before administration of the test substance.

After 15-20 min of equilibration, 0.1 mL of the extract or drug solution was injected intravenously followed by 0.1 ml of saline flush. BP was allowed to return to the resting level before every next dosing. Pulse pressure was obtained by subtracting diastolic BP (DBP) from systolic BP (SBP). Mean arterial blood pressure (MABP) was calculated by adding the values of DBP and one-third of pulse width. Change in blood pressure was recognized as the difference between the steady state values before and the lowest readings after administration of each dose of the test substance. 

*Angiotensin converting enzyme (ACE) inhibitory assay*

The activity of serum ACE was determined using hippuryl-l-histidyl-l-leucine (HHL) as the substrate. The enzyme hydrolyzes this molecule to give hippurate in the presence of boric acid/NaOH buffer containing NaCl. The final concentrations in the incubation mixture were 80 mmol/L boric acid (adjusted to pH 8.3 with 5 M NaOH), 800 mmol/L NaCl and 4 mmol/L of HHL. The liberated hippurate was reacted with the coloring reagent; *i.e. *cyanuric chloride/dioxane (9 g/L) in the presence of phosphate buffer (200 mmol/L, pH 8.3), to yield a chromogen, which was quantified from its absorbance at 382 nm (22). Rat blood was obtained through cardiac puncture under ketamine-diazepam anesthesia and allowed to clot for 15 min at room temperature; and the serum, separated by centrifugation for 15 min at the speed of 5000 rpm, was used as source of ACE.

Briefly, 0.1 mL of borate buffer was mixed with 0.05 mL of the extract solution and 0.05 ml of rat serum as source of ACE. After incubation at 37°C for 10 min, 0.05 mL of HHL solution (20 mM) warmed at 37°C was added to the reaction mixture and the reaction was allowed to proceed for 60 min at 37°C. The reaction was terminated with 0.25 mL of 1 M HCl solution and 30 sec later, neutralized with same volume of 1 M NaOH solution. Then 1 ml of phosphate buffer and 0.75 mL of coloring reagent were added, followed by mixing vigorously using vortex mixer (SeouLin Bioscience, Korea) for 30 sec in bursts of 5-10 sec, and allowed to stand for 5 min, vortex-mixed again, and then, centrifuged at 3000 rpm for 10 min in a bench centrifuge to remove denatured proteins and excess cyanuric chloride. Absorbance of the clear supernatant solution was measured by spectrophotometer (Model U2020, IRMECO Germany). For positive control, 50% DMSO was used instead of extract solution; and for negative control, terminating and neutralizing solutions were added just after serum before the substrate. Captopril (2 μM) was used as standard ACE inhibitor for comparison. All determinations were performed in triplicate. The percentage inhibition was calculated by using the formula: ACE Inhibition (%) = [(A _positive control_ - A _sample_) × 100] / [(A _positive control_ -A _negative control_)], where “A” is the absorbance of respective solution at 382 nm.

*Diuretic assay*

The diuretic assay was performed according to the method described by Jabeen *et al. *(23). Briefly, Sprague-Dawley rats were randomly assigned into five groups of five animals each. The control group received normal saline (10 mL/Kg, IP). Another group of animals was given frusemide (10 mg/Kg, IP) as standard diuretic. The treated groups of animals were injected with different doses of extract, intraperitoneally. Immediately after dosing, animals were individually housed in metabolic cages (Techniplast, Italy) and the urine was collected for 6 h. Total urine volume was calculated and Na^+^ and K^+^ urinary concentrations were measured by using clinical flame photometer (Model 410C, Sherwood, UK).

*Acute toxicity test*

Albino mice were divided into different groups of five animals in each group. Increasing doses of the extract (1, 2, 3 and 5 g/Kg) were given orally in 10 mL/Kg volumes, to different groups serving as test groups. Another group was given normal saline (10 mL/Kg) as negative control. The animals were allowed food and water *ad libitum *and kept under regular observation for 6 h and then lethality was noted after 24 h (23).

*Statistical analysis*

The results were analyzed statistically using software GraphPad Prism 5.01. The data was expressed as mean ± SEM. IC_50_ was calculated by nonlinear curve fitting. Student’s *t*-test was used to compare an experimental group with control group. The values at p < 0.05 were regarded as statistically significant.

## Results and Discussion

*Phytochemical analysis*

The extract was found positive for alkaloids, flavonoids, tannins and coumarins. Saponins, anthraquinones and cyanogenic glycosides were absent. Total phenolic contents as determined by Folin-Ciocalteu method were calculated as 152 ± 1.85 mg of gallic acid equivalent (GAE) per gram of the extract (n = 3). Presence of a variety of phytochemicals in the crude extract of *I. reniformis *(Ir.Cr.) indicates high therapeutic potential of the plant. The presence of phenolic compounds; especially tannins and flavonoids, suggests that the plant may have protective effects on cardiovascular system, as such compounds have been reported to have antioxidant, vasorelaxant, hypotensive and ACE inhibitory effects (24, 25). Short term oral administration of polyphenols has been shown to decrease blood pressure in rats via improvement in endothelium-dependant vasodilatations (26). 

*Hypotensive effect in anesthetized rats*

**Figure 1:**
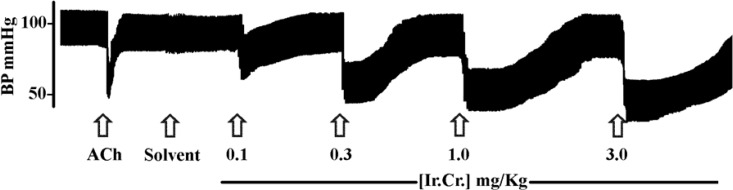
A typical tracing showing effects of acetylcholine (1 μg/Kg) and different doses of the aqueous-methanol extract of *Ipomoea reniformis *(Ir.Cr.) on blood pressure of an anesthetized rat. 5% DMSO in normal saline was used as solvent for Ir.Cr. Arrows indicate the time of administration of drugs

The intravenous administration of the crude extract of *I. reniformis *(Ir.Cr.) in anesthetized rats produced dose-dependant fall in mean arterial blood pressure (MABP), systolic blood pressure (SBP) and diastolic blood pressure (DBP) in the dose range of 0.1 to 3.0 mg/Kg. Figure 1 shows a representative tracing of an experiment and percent fall in MABP, SBP and DBP are summarized in Table 1.

**Table 1:** Effects of the crude extract of *Ipomoea reniformis *(Ir.Cr.) on systolic blood pressure (SBP), diastolic blood pressure (DBP) and mean arterial blood pressure (MABP) of anesthetized rats.

**Treatment (dose)**	**% Fall in SBP**	**% Fall in DBP**	**% Fall in MABP**
Ir.Cr. (0.1 mg/Kg)	10.08 ± 4.96	31.62 ± 4.00	21.51 ± 3.41
Ir.Cr. (0.3 mg/Kg)	18.49 ± 4.28	34.35 ± 2.20	28.99 ± 2.30
Ir.Cr. (1.0 mg/Kg)	38.55 ± 4.05	57.10 ± 3.89	53.34 ± 0.88
Ir.Cr. (3.0 mg/Kg)	51.44 ± 4.02	72.75 ± 2.83	61.71 ± 3.37

The values are mean ± SEM of four determinations

Maximum reduction in blood pressure was observed after administration of the extract (Ir.Cr.) at the dose of 3 mg/Kg, which is greater in magnitude and duration than the effect produced by acetylcholine at the dose of 1 μg/Kg (Figure 1). The extract decreased both SBP and DBP of the anesthetized rats but the fall in DBP was greater than that in SBP. This kind of blood pressure lowering effect has been observed with *β*-adrenergic receptor agonistic drugs, which cause vasodilation with increased cardiac output, resulting in greater fall of DBP (27). 

However, the involvement of other receptors and ion channels cannot be ignored. The extract was also found to decrease heart rate (HR), but the fall in HR was not dose-dependant, possibly due to the involvement of reflex mechanisms. Antihypertensive and hypotensive drugs may act by various mechanisms; such as direct and/or indirect effect on heart, relaxation and/or inhibition of contraction of vascular smooth muscle causing decrease in total peripheral resistance or combination of mechanisms. Results of our study demonstrated that the Ir.Cr. has hypotensive and cardiodepressent effects in anesthetized rats.

*Angiotensin Converting Enzyme (ACE) inhibition*

The crude extract of *I. reniformis *was tested for serum ACE activity *in-vitro *at the concentrations of 0.01, 0.025, 0.5, 1.0 and 2.0 mg/mL. The percentage inhibitions of serum ACE activity by different concentrations of the extract are shown in Table 2. The IC_50_ value of the extract was calculated as 422 ± 21.16 μg/mL (n = 3). Captopril, the standard ACE inhibitor, was found to inhibit serum ACE activity by 88.93 ± 3.78% at 2 μM concentration. 

**Table 2:** Angiotensin converting enzyme (ACE) inhibitory activity of the crude extract of *Ipomoea reniformis *(Ir.Cr.) and captoril, the standard ACE inhibitor

**Extract or drug (concentration)**	**% Inhibition of ACE activity**
Ir.Cr. (0.10 mg/mL)	27.13 ± 4.51
Ir.Cr. (0.25 mg/mL)	39.40 ± 3.18
Ir.Cr. (0.50 mg/mL)	55.86 ± 2.12
Ir.Cr. (1.00 mg/mL)	78.98 ± 3.16
Ir.Cr. (2.00 mg/mL)	81.11 ± 3.89
Captopril (2 μM)	88.93 ± 3.78

The values are mean ± SEM of three determinations

Angiotensin II, a decapeptide, plays important role in pathogenesis of hypertension, whose production depends upon the activity of ACE. Therefore, ACE inhibitors are one of the important therapeutic strategies for normalizing blood pressure in hypertensive patients. Several *in-vivo *and *in-vitro *studies have identified a range of medicinal plants which possess ACE inhibitory activity (28). Screening of these plants has identified several groups of natural ACE inhibitors including alkaloids, flavonoids, tannins, phenylpropanoids, proanthocyanidins, fatty acids, and terpenoids (24, 29, 30). ACE is a zinc metallopeptidase, which requires zinc ions for its activity. Tannins have general ability to precipitate water soluble proteins (31) and to chelate metal ions. The ACE inhibitory activity of the crude extract of *I. reniformis *may be due to its alkaloid, flavonoid and tannin contents, possibly through sequestration of enzyme metal co-factor, protein precipitation or through other mechanisms. 

*Diuretic effect*

The results of diuretic assay are summarized in Table 3. The volume of urine excreted by rats per 100 g of the body weight in 6 h for control group treated with normal saline (10 mL/Kg) was compared with other groups.

**Table 3:** Effects of the crude extract of *Ipomoea reniformis *(Ir.Cr.), normal saline (N.S.) and frusemide on urine output in Sprague-Dawley rats

**Group**	**Urine volume ** **(ml / 100 g / 6 h)**	**Na**+ **concentration** **(mmol/L)**	**K**+ **concentration (mmol/L)**	**Na**+ **/ K**+
N. S. (10 mL/Kg)	1.036 ± 0.136	49.67 ± 3.57	12.22 ± 0.77	4.00 ± 0.16
Frusemide (10 mg/Kg)	4.27 ± 0.047***	107.50 ± 4.31***	26.17 ± 2.57***	4.20 ± 0.24
Ir.Cr. (10 mg/Kg)	1.41 ± 0.08	52.33 ± 4.93	15.87 ± 1.50	3.40 ± 0.37
Ir.Cr. (30 mg/Kg)	1.89 ± 0.11*	69.83 ± 2.75**	15.58 ± 1.23*	4.70 ± 0.50
Ir.Cr. (50 mg/ Kg)	2.48 ± 0.26**	77.50 ± 7.71**	21.08 ± 1.55***	3.81 ± 0.32

The values are mean ± SEM of five determinations. p-values are compared with control (N.S.) group as *p< 0.05, **p< 0.01, ***p< 0.001

Frusemide (10 mg/Kg) was used as standard diuretic, which increased urine volume and urinary electrolytes significantly (p < 0.001), when compared to the saline treated control group. Intraperitoneal administration of the crude extract of *I. reniformis *at the doses of 10, 30 and 50 mg/Kg increased the urine volume in rats, but increase in urine and electrolytes output was not significant at the dose of 10 mg/Kg. However, the extract significantly increased urine volume and urinary electrolytes output at the doses of 30 and 50 mg/Kg compared to the control group. Further increase in dose did not increase the urine output in rats (data not shown). The diuretic effect of the extract was found less than that of the frusemide. The ratio Na^+^/K+ determines the natriuretic activity and value greater than 2 indicates favourable natriuretic effect (32). The Na^+^/K^+^ ratio of all the treated groups was calculated in the range of 3.40 ± 0.37 to 4.70 ± 0.50, suggesting the favourble natriuretic effect of the extract. 

Diuretics decrease plasma volume and subsequently venous return to the heart; *i.e*. preload. This decreases cardiac workload, oxygen demand and plasma volume, thus decreasing the blood pressure. Diuretics play an important role in volume overloaded and salt sensitive hypertensive patients (33). Previous studies demonstrated that several secondary metabolites of plants could be responsible for the diuretic effects, such as alkaloids, tannins, flavonoids, saponins, terpenoids or organic acids (34). Alkaloids and/or phenolic compounds detected in the Ir.Cr. may be considered as responsible for the diuretic activity of the medicinal plant. 

*Acute toxicity test*

In acute toxicity test, the Ir.Cr. was found to be safe at the tested doses of 1, 2, 3 and 5 g/Kg orally. 

## Conclusion

The results of this study show that the polyphenols rich crude extract of the dried aerial parts of *Ipomoea reniformis *(Ir.Cr.) has dose-dependant blood pressure lowering effect in rats. The extract also inhibited serum Angiotensin Converting Enzyme activity *in-vitro *with IC_50_ value of 422 ± 21.16 μg/mL. The extract was also found to have diuretic activity at the doses of 30 and 50 mg/Kg. The blood pressure lowering effect of the extract, at least in part, may be mediated through its ACE inhibitory and diuretic activities. Further studies are recommended to find out the involvement of other possible mechanisms in its blood pressure lowering activity. The present study justifies the traditional use of *Ipomoea reniformis *as blood pressure lowering and diuretic medicinal plant. 
